# The simple neuroendocrine-immune regulatory network in oyster *Crassostrea gigas* mediates complex functions

**DOI:** 10.1038/srep26396

**Published:** 2016-05-19

**Authors:** Zhaoqun Liu, Lingling Wang, Zhi Zhou, Ying Sun, Mengqiang Wang, Hao Wang, Zhanhui Hou, Dahai Gao, Qiang Gao, Linsheng Song

**Affiliations:** 1 Key Laboratory of Experimental Marine Biology, Institute of Oceanology, Chinese Academy of Sciences, Qingdao 266071, China; 2 University of Chinese Academy of Sciences, Beijing 100049, China; 3 Key Laboratory of Mariculture & Stock Enhancement in North China’s Sea, Ministry of Agriculture, Dalian Ocean University, Dalian 116023, China

## Abstract

The neuroendocrine-immune (NEI) regulatory network is a complex system, which plays an indispensable role in the immunity of the host. In the present study, the bioinformatical analysis of the transcriptomic data from oyster *Crassostrea gigas* and further biological validation revealed that oyster TNF (CgTNF-1 CGI_10018786) could activate the transcription factors NF-κB and HSF (heat shock transcription factor) through MAPK signaling pathway, and then regulate apoptosis, redox reaction, neuro-regulation and protein folding in oyster haemocytes. The activated immune cells then released neurotransmitters including acetylcholine, norepinephrine and [Met^5^]-enkephalin to regulate the immune response by arising the expression of three TNF (CGI_10005109, CGI_10005110 and CGI_10006440) and translocating two NF-κB (Cgp65, CGI_10018142 and CgRel, CGI_10021567) between the cytoplasm and nuclei of haemocytes. Neurotransmitters exhibited the immunomodulation effects by influencing apoptosis and phagocytosis of oyster haemocytes. Acetylcholine and norepinephrine could down-regulate the immune response, while [Met^5^]-enkephalin up-regulate the immune response. These results suggested that the simple neuroendocrine-immune regulatory network in oyster might be activated by oyster TNF and then regulate the immune response by virtue of neurotransmitters, cytokines and transcription factors.

The neuroendocrine-immune (NEI) regulatory network consists of nervous system, endocrine system and immune system, which carries a reciprocal regulation to maintain homeostasis in the host with the involvement of signaling molecules, such as neurotransmitters, hormones and cytokines^1^. The accumulating evidences in the last decades have clearly documented the vital importance of NEI network in the regulation of physiological homeostatic mechanisms, in particular with regard to immunomodulation functions^2^.

The structure and function of the NEI regulatory network in vertebrates have been well understood, and the connections between neuroendocrine system and immune system are mediated by nerve pathways, hormonal circuits, cytokines, neuropeptides and chemokines^3^. The central nervous system (CNS) mediates both innate and adaptive immunity through systemic, regional and local routes. The peripheral nervous system provides the first line of defense at local sites, while the sympathetic (or adrenergic) nervous system (SNS), and the parasympathetic (or cholinergic) nervous system generally inhibit inflammation at a regional level^4^. Once the host is invaded by pathogenic microorganisms, the immune system will be activated immediately by immune surveillance cells, and then generate immune mediators to activate peripheral sensory afferent neurons. The activated neurons release neurotransmitters, such as neuropeptides, to up-regulate the immune response level and transfer the immune signals to the CNS at the same time. When the immune response exceeds a certain threshold or the pathogens are completely removed, the CNS releases immune mediators such as glucocorticoids, acetylcholine and catecholamines by controlling adrenal or autonomic nervous system to suppress immune response in case of an immune injury^4^. Meanwhile, energy is re-allocated in the above processes to maintain the immune response and homeostasis^4^,^5^,^6^. There are various kinds of neuroendocrine-immune axes and neuroendocrine cells in mammals to impinge on neuro-immunomodulation^4^.

Analogous NEI network has also been found in invertebrates. The structure of nervous system in invertebrates is relatively simple, and its diversity and complexity increases along with the evolution^7^,^8^. A primitive nervous system consisting of two neurons exists in the most primitive multicellular organisms sponges, and there is neither synaptic contacts nor motion-control functions between neurons^9^. In Cnidaria, neurons contact with each other through synapses within a reticular nervous system^10^, while there is a trapezoidal nervous system in Platyhelminthes, indicating a dramatic progress in evolution^11^. A chain nervous system consisting of pharyngeal ganglia and abdominal ganglia occurs in Annelida and Arthropoda, dominating contractile activity of muscles^12^,^13^. Molluscs have the well-developed nervous system in invertebrates^14^ with the presence of cephalic ganglia, pleural ganglia, visceral ganglia and pedal ganglia^15^. For example, the nervous system of oyster *Crassostrea virginica* consists of two cerebral ganglia (CG) under the palps, and one visceral ganglia (VG) near the posterior adductor muscle with the nerve fibers connecting them^16^. Although the nervous systems vary dramatically in structure, a growing number of ancestral molecules have been found in invertebrates sharing similar roles in the immune regulation of vertebrates, which makes it believable that hormonal and neuronal immune-regulating routes do exist in invertebrates. Corticotropin-releasing hormone (CRH), biogenic Amine (BA), glucocorticoid (GC), adrenocorticotropic hormone (ACTH) and cytokines are considered as basic components of the immunomodulation in invertebrates^17^, while [Met^5^]-enkephalin (ENK), acetylcholine (ACh) and norepinephrine (NE) are present in an extensive range of the phylum. However, the knowledge about the activation mechanism of neuroendocrine system and its regulation in the invertebrate immune response is still very limited.

The molluscs have evolved a primitive neuroendocrine system and a sophisticated immune system, which are more complex and important than what we have learned^18^. In the previous studies, catecholaminergic and cholinergic system, [Met^5^]-enkephalin and its receptor, as well as cytokines such as tumor necrosis factor (TNF) were reported to be existed in scallop *C. farreri*^19^,^20^,^21^ and oyster *C. gigas*^22^,^23^, which opened up new horizons and provided us an entrance to a better understanding of the neuroendocrine-immune regulatory network in invertebrates. The information of the NEI regulatory network in mollusc will contribute to the understanding of the NEI system evolution, and also throw light on the study of adaptation of invertebrates to the environment. The purposes of this study are to (1) identify the molecular components of NEI regulatory network in oyster *C. gigas*, (2) explore the potential activation mechanism of neuroendocrine system, (3) investigate the immunomodulation of neurotransmitter acetylcholine, norepinephrine and [Met^5^]-enkephalin, and (4) evaluate the synergic effects of neurotransmitters on oyster immune system.

## Results

### The output of RNA-Seq sequencing

Sixteen transcriptome fragment libraries were constructed with RiboMinus RNA from oyster haemocytes in eight groups, and each group had two parallel libraries. These libraries were sequenced using SOLiD 4 Genetic Analysis System, and a total of 1,019,503,323 single end reads with length of 50 bp were obtained. The read numbers in each library were shown in Table 1, and the combined sequence length was over 50,975 Mbp, representing 91.4-fold coverage of the oyster genome size.

After filtration with the sequences of adapter, Poly-N and known non-coding RNAs, 14,001 reads were removed. The remaining clean reads in each library were mapped to the oyster genome, and the successfully mapping rates ranged from 32.80% to 59.24% (Table 1). The uniquely mapping rates from sixteen libraries fluctuated within the relatively small range (from 68.63% to 73.17%, Table 1).

### Differentially expressed genes after CgTNF-1 stimulation and their GO annotation

**Figure 1:**
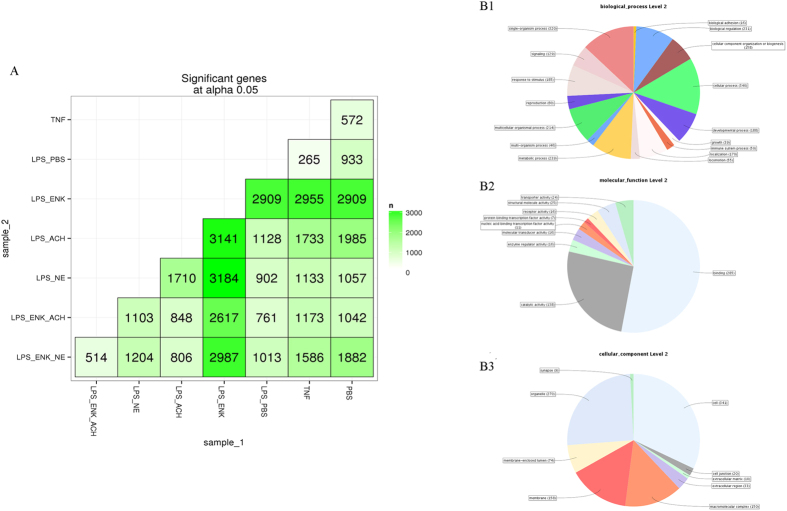
The overall expression level of all the genes and the differentially expressed genes between control and experiment groups. (**A**) The matrix of differentially expressed genes between any two groups. (**B**) Sequence distribution by GO at level 2 for differentially expressed genes between the TNF and PBS groups (**B1**: Biological process; **B2**: Molecular function; **B3**: Cellular component). Numbers shown in the chart represented the amount of sequences with corresponding GO terms.

The genes in oyster haemocytes from the eight treatment groups shared similar expression level, excepted for the relatively lower level in the LPS_ENK group (figure not shown). There were 572 genes differentially expressed in CgTNF-1 group in comparison to PBS group (Fig. 1A).

The GO functional analysis of the differentially expressed genes in the comparison of TNF versus PBS group was applied. A total of 572 differentially expressed genes including 191 up-expressed genes and 381 down-expressed genes were assigned with GO terms (level-2), which were summarized into three main categories: biological process, molecular function and cellular component (Fig. 1B). In biological process category, the top-3 GO terms were cellular process (GO: 0009987, 13.8%), single-organism process (GO: 0044699, 13.0%) and metabolic process (GO: 0008152, 9.7%) (Fig. 1B1). In molecular function category, binding (GO: 0005488, 52.9%), catalytic activity (GO: 0003824, 25.6%) and structural molecule activity (GO: 0005198 4.6%) accounted for the major portion (Fig. 1B2). And the cellular component category mainly consisted of cell (GO: 0005623, 32.0%), organelle (GO: 0043226, 25.3%) and membrane (14.8%) (Fig. 1B3).

### The TNF regulatory network in oyster

**Figure 2:**
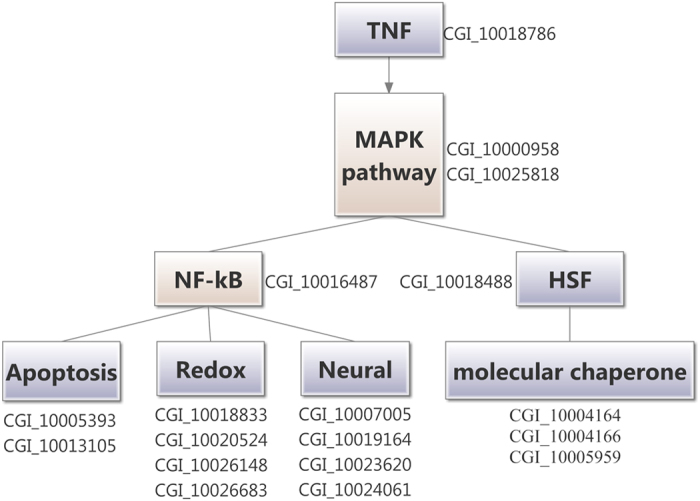
The deduced regulatory network of TNF (CGI_10018786). The network comprised signal pathway, transcription factor and effectors.

Two MAPK pathway-related genes, one NF-κB and one HSF were sorted out as differentially expressed genes between the PBS and TNF group. Furthermore, two apoptosis-related genes, four redox system-related genes, four neural genes and three molecular chaperone-related genes were parsed by hand. Based on the present results and the prior knowledge, a possible regulatory network of cytokines and transcription factors in oyster was outlined in Fig. 2, in which TNF (CGI_10018786) triggered the activation of transcription factors NF-κB and HSF through the activation of MAPK signal pathway, and then regulated the apoptosis (CGI_10005393: inhibitor of apoptosis protein, CGI_10013105: apoptosis 2 inhibitor), redox reaction (CGI_10018833: extracellular superoxide dismutase [Cu-Zn], CGI_10020524: calcium-binding protein, CGI_10026148: extracellular superoxide dismutase [Cu-Zn], CGI_10026683: dual oxidase), neuro-regulation (CGI_10007005: estrogen-related receptor gamma, CGI_10019164: FMRFamide receptor, CGI_10023620: neuromedin-U receptor 2, CGI_10024061: DBH-like monooxygenase protein 1-like protein) and protein folding (CGI_10004164: stress-induced protein 1, CGI_10004166: stress-induced protein, CGI_10005959: alpha-crystallin B chain) in oyster haemocytes.

### Differentially expressed genes after neurotransmitter stimulation and their GO annotation

**Figure 3:**
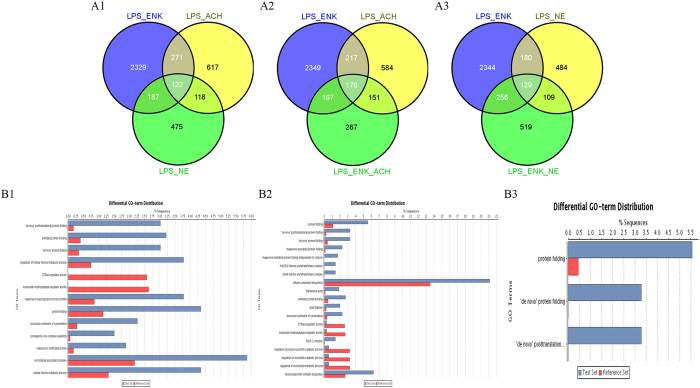
Grouping and enrichment analysis of differentially expressed genes. (**A1**) The grouping of the gene lists of LPS_ENK, LPS_ACh and LPS_NE. (**A2**) The grouping of the gene lists of LPS_ENK, LPS_ACh and LPS_ENK_ACh. (**A3**) The grouping of the gene lists of LPS_ENK, LPS_NE and LPS_ENK_NE. (**B1**) Enrichment analysis result between the gene lists of LPS_ENK_ACh and LPS_ENK. (**B2**) Enrichment analysis result between the gene lists of LPS_ENK_NE and LPS_ENK. (**B3**) Enrichment analysis result between the gene lists of LPS_ENK_NE and LPS_NE.

Five differentially expressed gene lists were obtained in the comparison of the samples LPS_ENK versus LPS_PBS, LPS_ACh versus LPS_PBS, LPS_NE versus LPS_PBS, LPS_ENK_ACh versus LPS_PBS, and LPS_ENK_NE versus LPS_PBS.There were 2,909, 1,128, 902, 761 and 1,013 differentially expressed genes in each list, respectively. Furthermore, there were 122 differentially expressed genes shared in the gene lists of the LPS_ENK group, LPS_ACh group and LPS_NE group, while 2,329, 617 and 475 differentially expressed genes were only found in the gene lists of LPS_ENK, LPS_ACh and LPS_NE group, respectively (Fig. 3A1). There were 267 differentially expressed genes in the gene list of LPS_ENK_ACh group, which were not included in the gene lists of LPS_ENK and LPS_NE group (Fig. 3A2). There were 519 differentially expressed genes included in the gene list of the LPS_ENK_NE group, while not in the gene lists of LPS_ENK and LPS_ACh group (Fig. 3A3).

The enrichment analysis of GO terms between the gene lists of LPS_ENK_ACh and LPS_ENK group was completed at multiple GO levels (Fig. 3B1). The three major enriched GO terms in Biological Process category were ‘de novo’ posttranslational protein folding (GO: 0051084, over, FDR < 2.89E-04), ‘de novo’ protein folding (GO: 0006458, over, FDR < 3.61E-03) and regulation of cellular ketone metabolic process (GO: 0010565, over, FDR < 1.0E-02). The enriched GO items included response to topologically incorrect protein (GO: 0035966, over, FDR < 2.0E-02), protein folding (GO: 0006457, over, FDR < 3.03E-2), proteasome core complex assembly (GO: 0080129, over, FDR < 4.82E-02), misfolded protein (GO: 0051788, over, FDR < 4.82E-02), and cellular ketone metabolic process (GO: 0042180, over, FDR < 4.82E-02). In the Molecular Function category, the enriched GO terms were unfolded protein binding (GO: 0051082, over, FDR < 3.61E-03), GTPase regulator activity (GO: 0030695, under, FDR < 1.42E-02), nucleoside-triphosphatase regulator activity (GO: 0060589, under, FDR < 1.42e-02) and structural constituent of cytoskeleton (GO: 0005200, over, FDR < 4.81E-02). In addition, microtubule associated complex (GO: 0005875, over, FDR < 4.82E-02) was enriched in the category of Cellular Component. There was no enriched GO term between the gene lists of LPS_ENK_ACh and LPS_ACh group.

The distributions of enriched GO term between the gene lists of LPS_ENK_NE and LPS_ENK group were shown at multiple GO levels in Fig. 3B2. There were seven enriched GO terms including ‘de novo’ posttranslational protein folding, ‘de novo’ protein folding, protein folding, unfolded protein binding, GTPase regulator activity, nucleoside-triphosphatase regulator activity and structural constituent of cytoskeleton which were also enriched between the gene lists of LPS_ENK_ACh and LPS_ENK group, and other enriched GO terms were different between the two enrichment analysis. Meanwhile, there were three enriched GO terms between the gene lists of LPS_ENK_NE and LPS_NE group which were comprised of protein folding (GO: 0006457, over, FDR < 5.53E-03), ‘de novo’ protein folding (GO: 0006458, over, FDR < 0.03.60E-02) and ‘de novo’ posttranslational protein folding (GO: 0051084, over, FDR < 0.03.60E-02) in the Biological Process category (Fig. 3B3).

### The clustering of differentially expressed immune-related genes after neurotransmitter stimulation in the immune process

**Figure 4:**
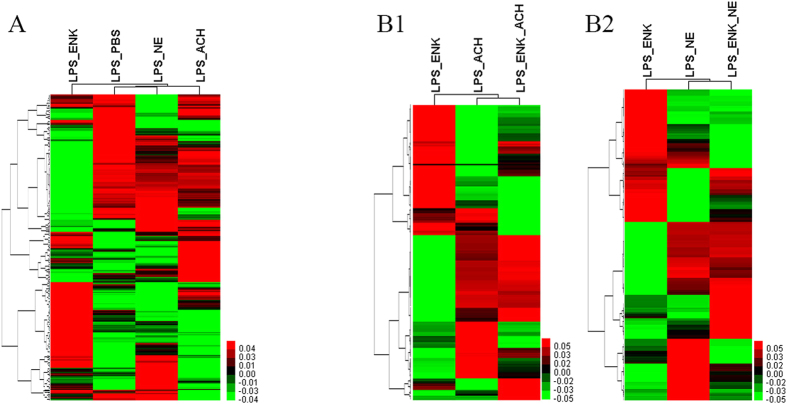
Cluster map of differentially expressed immune-related genes. (**A**) Summary cluster map of differentially expressed immune-related genes among the gene lists of LPS_ENK, LPS_ACh and LPS_NE. (**B1**) Summary cluster map of differentially expressed immune-related genes among the gene lists of LPS_ENK, LPS_ACh and LPS_ENK_ACh. (**B2**) Summary cluster map of differentially expressed immune-related genes among the gene lists of LPS_ENK, LPS_NE and LPS_ENK_NE.

After the merging of LPS_ENK, LPS_ACh, and LPS_NE gene lists, there were 361 differentially expressed immune-related genes annotated as the term of immune system process (GO:0002376, level-2) in the Biological Process category. The expression level of these 361 genes was retrieved, and applied to the clustering analysis. The expression profile of these immune-related genes in the LPS_ENK group was completely different from that in the LPS_PBS group. And the immune-related genes from the LPS_NE group displayed more similar expression profile with that from the LPS_PBS group than that from the LPS_ACh group (Fig. 4A).

In the same way, clustering analysis of differentially expressed immune-related genes in the LPS_ENK, LPS_ACh, and LPS_ENK_ACh groups was performed, and their expression profiles in the LPS_ENK_ACh group were found to be more similar with that in the LPS_ACh group (Fig. 4B1). Meanwhile, the immune-related genes in LPS_ENK_NE group were clustered firstly with those in the LPS_NE group (Fig. 4B2).

### The co-expression network under different neurotransmitter stimulation

**Figure 5:**
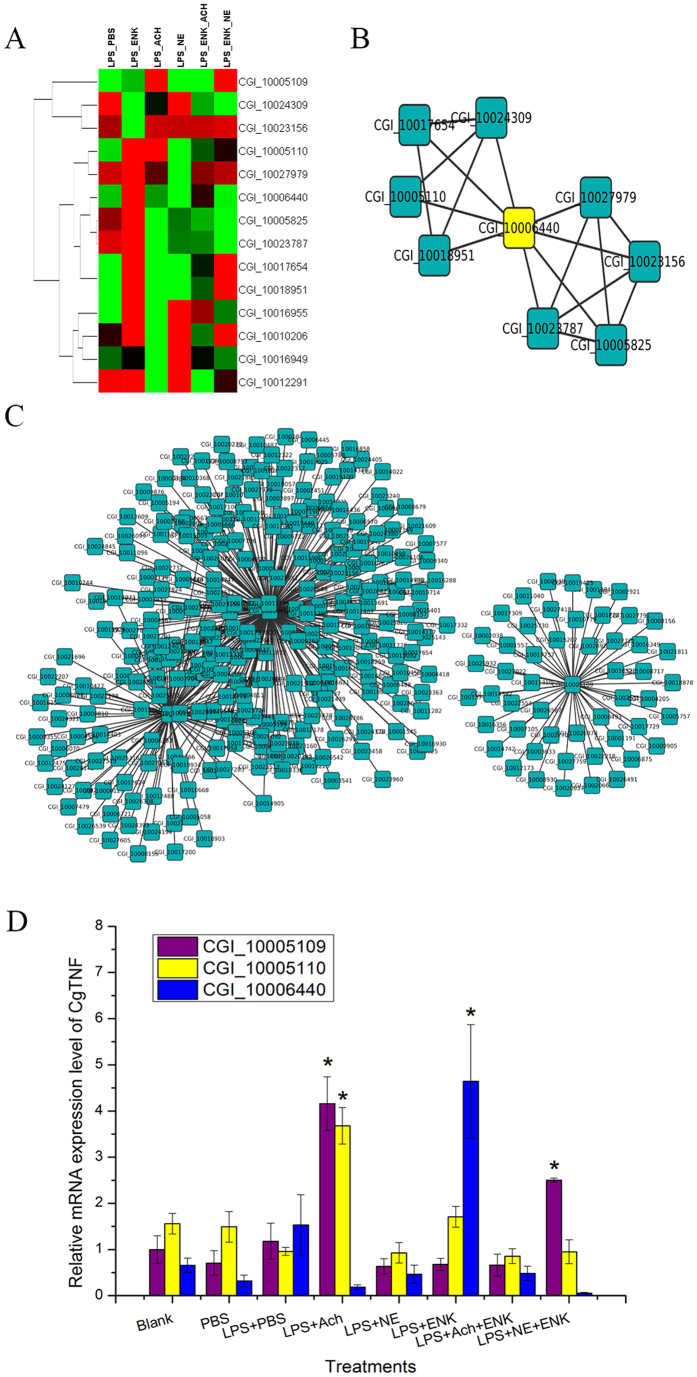
The expression pattern of cytokines and transcription factors after neurotransmitter treatment. (**A**) Fourteen differentially expressed genes in the TNF, NF-κB and AP-1 pathway and their expression level in the six groups. (**B**) The network that shows the internal connectivity among the 14 genes in the turquoise module. (**C**) The co-expression network of 3 TNF genes indicates their direct connectivity with other immune-related genes. (**D**) The relative mRNA expression level of 3 TNF genes in the eight groups.

To verify the activating function of TNF, 14 differentially expressed genes in the TNF, NF-κB and AP-1 pathways were retrieved to investigate the internal connectivity and co-expression network. The information in gene lists and co-expression network were listed in the Table 2 and shown in Fig. 5A. For the internal connectivity among the 14 genes, 4 genes (CGI_10005110: tumor necrosis factor ligand superfamily number 14, CGI_10017654: NF-kappa-B inhibitor epsilon, CGI_10018951: AP-1 complex subunit gamma-1 and CGI_10024309: heat shock protein 75 kDa) in the yellow module and 5 genes (CGI_10006440: tumor necrosis factor ligand superfamily member 11, CGI_10005825: tripeptidyl-peptidase 2, CGI_100023787: AP-2 complex subunit beta, CGI_10023156: TNF receptor-associated factor 3 and CGI_10027979: TNF receptor-associated factor 6) in the turquoise module formed a network with TNF (CGI_10006440) as the hubgene (Fig. 5B). There was no internal connectivity among other 5 genes. For the co-expression network of three TNF genes (CGI_10005109, CGI_10005110 and CGI_10006440), their direct connections with other genes were shown in Fig. 5C. A total of 51 immune-related genes were connected with CGI_10005109, while 100 immune-related genes were connected with CGI_10005110, and 228 immune-related genes were connected with CGI_10006440. The network of CGI_10005110 and CGI_10006440 joined together, whereas the network of CGI_10005109 was separated.

### The change of TNF mRNA expression after neurotransmitter stimulation

The mRNA expression of three TNF genes in oyster haemocytes at 6 h after neurotransmitter stimulation was determined by quantitative real-time PCR (Fig. 5D). The mRNA expression levels of CGI_10005109 in the groups of LPS_ACh and LPS_NE_ENK increased significantly by 4.16-fold and 2.50-fold (*p* < 0.05) respectively in comparison with that in the blank group. While the mRNA expression level of CGI_10005110 increased dramatically by 3.70-fold (*p* < 0.05) after LPS_ACh stimulation. Moreover, the mRNA expression level of CGI_10006440 in the LPS_ENK group increased significantly by 4.64-fold (*p* < 0.05) compared to that in the blank group. No significant difference was observed in other groups after neurotransmitter stimulation.

### Translocation of NF-κB in cytoplasm and nuclei of haemocytes after neurotransmitter stimulation

**Figure 6:**
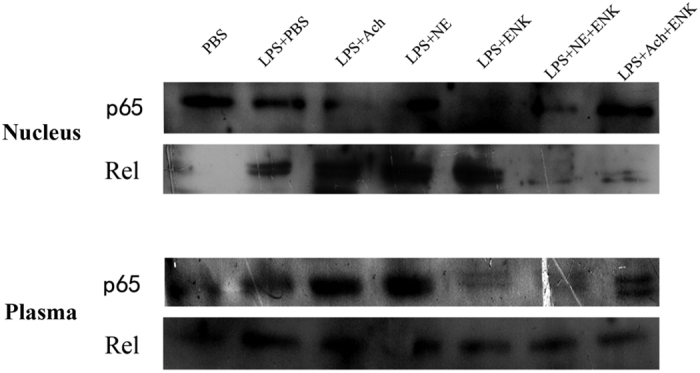
The translocation of 2 oyster NF-κB molecules in the nuclei and cytoplasm of haemocytes. Cropped blots are shown in the main figure, and the full-length blots are included in the Supplementary Fig. S1.

In order to explore the process of transcription factors getting in and out of nucleus after neurotransmitter treatment, the concentrations of two NF-κBs (Cgp65, 49.7 kD and CgRel, 23.0 kD) were determined by western blotting (Fig. 6 and Fig. S1). The concentration of Cgp65 in nuclei was significantly lower than that in cytoplasm in LPS_ACh and LPS_NE group, while it was much higher in nuclei than that in cytoplasm in LPS_PBS, LPS_ENK, LPS_ACh_ENK and LPS_NE_ENK group. As for CgRel, the concentration in nuclei was obviously lower than that in cytoplasm in LPS_ACh_ENK and LPS_NE_ENK group, while there was no significant difference of concentration between cytoplasm and nuclei in the other groups.

### Apoptosis index and phagocytosis rate of oyster haemocytes after neurotransmitter stimulation

**Figure 7:**
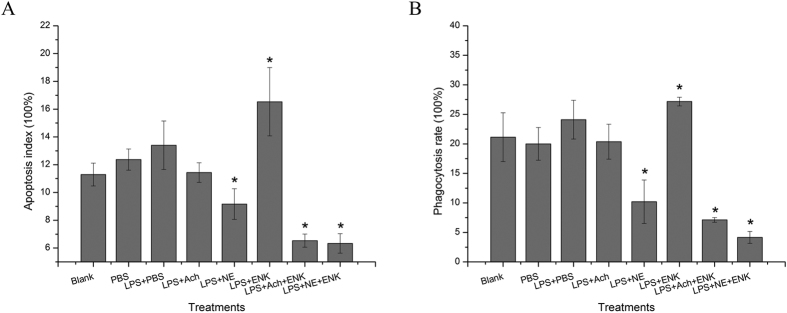
The apoptosis index (**A**) and phagocytosis rate (**B**) of oyster haemocytes in the eight groups.

To further validate the immunomodulation function of the neurotransmitters, the apoptosis index and phagocytosis rate of oyster haemocytes after neurotransmitter stimulation were determined by flow cytometry (Fig. 7). The apoptosis index of oyster haemocytes in LPS_ACh and LPS_NE groups decreased at 6 h after stimulation but the significant difference was only observed in LPS_NE group with the apoptosis index decreasing from 11.35% to 9.17% (*p* < 0.05) compared to that in the blank group. Similar trends were also observed in LPS_ACh_ENK and LPS_NE_ENK groups since the apoptosis index decreased dramatically from 11.35% to 6.53% and 6.33%, respectively, compared to that in the blank group (*p* < 0.05) (Fig. 6A). However, the injection of [Met^5^]-enkephalin induced the significant increase of apoptosis rate to 15.53% (*p* < 0.05). Meanwhile, the phagocytosis rate of oyster haemocytes in LPS_ACh, LPS_ACh_ENK and LPS_NE_ENK groups decreased dramatically from 21.33% to 10.20%, 7.13% and 4.17% separately in comparison to that in the blank group (*p* < 0.05). The phagocytosis rate in LPS_ENK group increased significantly from 21.33% to 27.17% compared to that in the blank group (*p* < 0.05) (Fig. 6B).

## Discussion

The communication and interaction between neuroendocrine and immune system is an important regulatory mechanism for the maintenance of immune homeostasis in vertebrate, and it also plays important role in the immunomodulation of some invertebrates^24^,^25^. In the present study, the molecular features, activation mechanism, and the immunomodulation roles of NEI system were investigated to better understand the NEI regulatory network in oyster *C. gigas*.

As the key molecules of the NEI regulatory network, cytokines are involved in most of the immune processes, including pathogenesis, non-specific response to infection and specific response to antigen^26^. Several cytokines, such as IL-1, IL-2, IL-6, TNF-α and IFN, have been reported to regulate the release of pituitary hormones by an action on the hypothalamus and/or the pituitary gland in vertebrate^27^. It has been reported that the hormone secretion will alternate when the host is infected, and cytokines may play roles in these alterations^27^. In the present transcriptome analysis, the NEI system in oyster could be quickly activated by tumor necrosis factor (CgTNF-1). After CgTNF-1 treatment, two MAPK pathway-related genes, one NF-κB gene and one HSF gene were observed to be differentially expressed. In addition, two apoptosis-related genes, four redox system-related genes, four neural genes and three molecular chaperone-related genes were also sorted out as differentially expressed genes. Subsequently, a regulatory network predominated by CgTNF-1 was established, in which CgTNF-1 might trigger transcription factor NF-κB and HSF through the activation of MAPK signal pathway, and then regulate the apoptosis, redox reaction, neuro-regulation and protein folding in oyster haemocytes. It was suspected that the oyster neuron was activated by CgTNF-1, and the activated neurons would release neurotransmitters to modulate oyster haemocyte through the MAPK pathway. Moreover, in the co-expression network, four genes (CGI_10005110, CGI_10017654, CGI_10018951 and CGI_10024309) and other five genes (CGI_10006440, CGI_10005825, 100023787, CGI_10023156 and CGI_10027979) in the turquoise module formed a network with TNF (CGI_10006440) as the hubgene. And the two networks separately taking TNF gene (CGI_10005110 and CGI_10006440) as hubgenes joined together. It indicated that oyster TNF could activate the immune system with the involvement of NF-κB family, which was similar to that in vertebrates. Although the precise mechanism of this activation is still unclear, a signaling pathway mediated by TNF and NF-κB seems to be the dominant way of oyster neuroendocrine system to trigger the immune response. The present results suggested that oyster TNF might share similar mechanism with vertebrate TNF to initiate immune response through MAPK signaling pathway, and then probably modulate the expression of hundreds of immune-related genes.

After the immune system is activated, the immune cells will release cytokines to activate some signaling pathways and subsequently regulate the synthesis and secretion of different neurotransmitters including dopamine, norepinephrine, acetylcholine and enkephalin^28^. For instance, catecholamines, as neurotransmitter and neuromodulator in nervous system, can modulate the innate and adaptive immunity of vertebrates^29^,^30^,^31^. Acetylcholine released from the activated cholinergic nervous system of mammals can regulate the level of intracellular reactive oxygen species (ROS) by cGMP-dependent protein kinase (PKG) pathway and transactivation of epidermal growth factor receptors (EGFRs)^32^,^33^. In the present study, three typical neurotransmitters, acetylcholine (ACh), norepinephrine (NE) and [Met^5^]-enkephalin (ENK), were employed in the stimulation experiment, and the expressional alternations of immune-related genes were recorded by RNA-seq technique. The mRNA expression levels of CGI_10005109 in LPS_ACh and LPS_NE_ENK groups increased significantly, while those of CGI_10005110 and CGI_10006440 increased dramatically in LPS_ACh group and LPS_ENK group, respectively. The higher expression level of these three TNFs might be partly due to the requirements of their participations in the immune regulation by neurotransmitters. These results indicated that the TNF family was not only the key molecules to activate the immune system, but also vital components of the signaling pathway for the neurotransmitter to modulate the endocrine system of oyster.

In vertebrates, TNF can bind to TNF receptors (TNFRs) expressed on both glia and neurons^34^. The expression of TNF gene is subjected to autoregulation via the activated NF-κB^35^, which is latent in the cytoplasm and bound to an inhibitory κB protein (IκB)^36^. Once IκB is degraded, NF-κB will be allowed to enter into the nucleus and exert its transcriptional activity for the genes including NOS, neurotransmitters, cytokines, immunoglobulin, IκB, and Rel families^37^. The activation of NF-κB is triggered by the rise of intracellular calcium, glutamate, ultraviolet light, protein kinase, oxidative stress, and cytokines such as IL-1 and TNF-а^38^,^39^,^40^. In the present study, the mRNA transcript of TNF after neurotransmitter stimulation was detected by quantitative real-time PCR to further understand the possible regulation of neurotransmitters on immune system in oysters. When the neuroendocrine-immune system of oyster was activated by TNF (CGI_10018786) through MAPK signaling pathway with the involvement of NF-κB (Cgp65 and CgRel), the activated neurons and immune cells released neurotransmitters, neuromediators and neuromodulators to regulate the immune system by promoting the mRNA expression level of TNF (CGI_10005109, CGI_10005110 and CGI_10006440) in the haemocytes. The expression levels of CGI_10005109 and CGI_10005110 increased significantly in LPS_ACh group, while CGI_10005109 was significantly overexpressed in the LPS_NE_ENK group. In addition, the expression level of another oyster TNF CGI_10006440 was dramatically increased in the LPS_ENK group. It is reported that neurotransmitters accomplish immunomodulation with the involvement of transcription factors. In the present study, the concentration differences of Cgp65 and CgRel between the cytoplasm and the nuclei reflexed the translocation of NF-κB molecules from cytoplasm into the nucleus, where they bound to immune-related sequences of DNA and then recruited other proteins such as coactivators and RNA polymerase to initiate the transcription and finally resulted in a change of cell function. In the neurotransmitters stimulation groups, the concentration of Cgp65 in nuclei was lower than that in the cytoplasm, indicating that neurotransmitters could regulate immune response by inhibiting the transcription mediated by Cgp65. On the opposite, there was no significant change of CgRel concentration in other groups. The above results suggested that the TNF family could activate the neuroendocrine system, and participate in the immunomodulation of the neurotransmitters.

After immune stimulation, the neuroendocrine system releases hormones, neurotransmitters and neuromediators to modulate the behavior of immunocytes, which can recognize and eliminate the invaders such as bacteria, viruses, *etc*^41^. Apoptosis is an important mechanism for the adequate clearance of infected, damaged and exhausted cells, especially when the hosts suffer from infection or dissemination^42^. Phagocytosis is an indispensable cellular mechanism to recognize and ingest non-self molecules and cell debris. It includes the process of phagocytic receptor recognition and the signaling pathway activation, which would lead to the dramatic changes in the dynamics of plasma membrane and cytoskeleton. Previous studies have suggested that phagocytosis could be influenced by neuropeptides^43^. In the present study, the apoptosis index and the phagocytosis rate were assayed with flow cytometry after the oysters were treated by acetylcholine, norepinephrine and [Met^5^]-enkephalin. The apoptosis index of oyster haemocytes in the groups of LPS_ACh (11.43%, *p* < 0.05) and LPS_NE (9.17%, *p* < 0.05) both decreased significantly after stimulation, while it increased in the LPS_ENK (16.53%, *p* < 0.05) group. The phagocytosis rate of oyster haemocytes was similar to the apoptosis index, except that the haemocytes phagocytosis rate in the LPS_NE (10.20%, *p* > 0.05) group did not change significantly compared with that in PBS and LPS group after neurotransmitter stimulation. The results indicated that acetylcholine and norepinephrine tended to down-regulate the cellular immune response, while [Met^5^]-enkephalin up-regulated the cellular immune response of oysters. It was suspected that different types of neurotransmitters could regulate the immune system in different ways, resulting in either up-regulation or down-regulation of the immune response.

The neuroendocrine-immune regulatory network functions through an extremely complicated mechanism, and the interactions among different neurotransmitters could induce various immune responses and diseases. For example, the imbalance between cholinergic activity and dopaminergic activity in the striatum causes a variety of neurological disorders, such as Parkinson’s disease^44^. Understanding the mechanisms of the synergistic regulation of different neurotransmitters may offer us some indications about the real biological process. In the present study, only one TNF (CGI_10005109) was up-regulated when the oyster was co-treated with norepinephrine and [Met^5^]-enkephalin, indicating that there might be some other molecules involved in the regulation of the immune response under the co-stimulation of neurotransmitters. In both LPS_ACh_ENK and LPS_NE_ENK groups, the concentration of Cgp65 in cytoplasm was significantly lower than that in the nuclei, indicating a translocation of Cgp65 from the cytoplasm into the nuclei after co-stimulation of ACh_ENK or NE_ENK. However, CgRel was transferred oppositely from the nuclei into the cytoplasm after co-stimulation. The above results indicated that the transcription regulation induced by co-stimulation of neurotransmitters was likely mediated mainly by transcription factor Cgp65. Furthermore, the apoptosis index and the phagocytosis rate were assayed with flow cytometry to investigate the synergistic physiologican effects of neurotransmitters. It has been reported that [Met^5^]-enkephalin can significantly up-regulate haemocyte phagocytosis rate and apoptosis index, while norepinephrine is able to modulate haemocyte ROS level and phagocytosis via β-adrenergic receptor in oyster *C. gigas*^23^,^43^,^45^. In the present study, both apoptosis index and phagocytosis rate of oyster haemocytes decreased significantly when acetylcholine and norepinephrine were separately injected into oysters together with [Met^5^]-enkephalin, suggesting that the activity of [Met^5^]-enkephalin to up-regulate the immune response could be overwhelmed by the down-regulation effects of acetylcholine and norepinephrine. These results collectively suggested that there might exist a primitive synergistic neuroendocrine-immune regulatory network in oyster, in which the immune cells were more sensitive to acetylcholine and norepinephrine than [Met^5^]-enkephalin.

The activation and immunomodulation are two major processes of neuroendocrine-immune regulatory network during the immune response of oyster. In conclusion, the neuroendocrine system of oyster could be activated by CgTNF-1 through MAPK signaling pathway with the involvement of NF-κB and HSP. Oyster neurons might be activated and then release neurotransmitters to modulate the immune response by promoting the expression of TNF and translocation of NF-κB in cytoplasm and nuclei of haemocytes. The results provided insights into mechanisms of neuroendocrine-immune regulatory network in oysters and highlighted oysters as the potential model for addressing the evolutionary implications of neuroendocrine-immune system.

## Methods

### Oysters, TNF protein and neurotransmitter treatment

Oysters *C. gigas* with averagely 110 mm in shell height were collected from a local farm in Qingdao, Shandong Province, China, and maintained in the aerated seawater at 20 °C for two weeks before processing.

Two hundred and eighty oysters were employed and averagely divided into eight groups. Oysters in two groups received an injection of 100 μL phosphate buffered saline (PBS, 0.14 M sodium chloride, 3 mM potassiumchloride, 8 mM disodium hydrogen phosphatedodecahydrate, 1.5 mM potassium phosphate monobasic, pH 7.4, designated as PBS group) and CgTNF-1 protein (10 ng mL^−1^, prepared according to Sun *et al.*^22^, designated as TNF groups), respectively. Oyster in the remaining six groups received injections of 100 μL of *E. coli* PBS (commercially purchased, designated as LPS_PBS group), [Met^5^]-enkephalin (10^−4 ^mol L^−1^ in PBS^46^, designated as LPS_ENK group), acetylcholine (10^−7 ^mol L^−1^ in PBS^47^, designated as LPS_ACh group), norepinephrine (10^−6 ^mol L^−1^ in PBS^21^, designated as LPS_NE group), mixture 1 (10^−4 ^mol L^−1^ ENK and 10^−7 ^mol L^−1^ ACh in PBS, designated as LPS_ACh_ENK group), and mixture 2 (10^−4 ^mol L^−1^ ENK and 10^−6 ^mol L^−1^ NE in PBS, designated as LPS_NE_ENK group) at 6 h after the injection of 100 μL lipopolysaccharide (LPS, in PBS), respectively. Oysters without any treatments were designated as the blank group. After treatment, these oysters were returned to water tanks, and sampled at 6 h post-injection. Haemolymph from ten individuals in each group were collected from the blood sinus with an injector and pooled together as one sample and centrifuged at 800 g at 4 °C for 10 min to harvest the haemocytes. Each sample was adjusted with equal haemocyte numbers. Haemocyte samples were stored at −80 °C after addition of 1 mL Trizol reagent (Invitrogen) for subsequent RNA extraction and library preparation.

### Library preparation

Total RNA was isolated from haemocytes using Trizol reagent (Invitrogen) according to its protocol. The extracted RNA was quantified by Nanodrop 2000 (Thermo Scientific) and checked for the integrity with Angilent 2100 Bioanalyzer (Agilent Technologies). The mRNA was purified with Dynabeads® mRNA DIRECTTM Micro Kit (Invitrogen) following the manufacturer’s instructions.

The single-end fragment library was constructed following the SOLiD Total RNA-Seq Kit protocol (Life Technologies, PN4452437). Ribo-minus RNA was fragmented by RNase III and purified by the RiboMinus Concentration Module (Invitrogen). RNA fragment was linked with the adaptors using the hybridization master mix (SOLiD Total RNA-Seq Kit), and reverse transcription was performed subsequently. The purified cDNA was size-selected after electrophoresis with the Novex TBE-Urea Gel (Invitrogen) at 180 V for 20 min according to Wang *et al.*^48^. The gel block containing 150–250 nt cDNA was precisely excised and used as amplification template. The PCR reactions were performed at 95 °C for 5 min and then cycled at 95 °C for 30 sec, 62 °C for 3 sec and 72 °C for 30 sec for 16 cycles in a thermal cycler. All of the components used in the amplification were from the SOLiD Total RNA-Seq Kit. The yield and size distribution of PCR products were checked by Agilent 2100 Bioanalyzer.

Emulsion PCR and bead enrichment were performed using the SOLiD EZ Bead^TM^ system (Life Technologies). Workflow analysis (WFA) was performed firstly to verify the quality and density of the template beads, and the enriched beads for each sample were then deposited on the sequencing slide. Finally, the libraries were sequenced by the SOLiD 4 sequencing platform and color-space reads were outputted. The raw sequencing reads were submitted to NCBI Short Read Archive under the accession number of SRA.

### Analysis of differentially expressed transcripts

Oyster genomic sequences and annotation file are available at the Comprehensive Library for Modern Biotechnology (CLiMB) repository (doi:10.5524/100030). Bioinformatics analysis was performed according to the description of Wang *et al.*^48^. Briefly, the reads alignment was performed using LifeScopeTM software (Life Technologies) with default parameters. The classic mapping strategy “seed-and-extend” approACh was adopted, with “25.2.0:20” as mapping scheme (for the 50 base reads, the seed might be 25 base long with up to two mismatches allowed, and the start site of seed could be 0 or 20). Cufflinks was used to assemble transcripts, estimate their abundances and identify the differentially expressed transcripts between the stimulation and control groups. The overall situation of RNA-Seq was analyzed using the CummeRbund an R package.

### GO analysis and KEGG

The GO enrichment analysis was implemented by the one-tailed Fisher’s exact test with filter value set as 0.001. The differentially expressed genes were selected as test set while all identified transcripts were taken as the reference set. The significantly over-enriched GO in test set was reported, and the differentially expressed genes were mapped to the KEGG database, using built-in function of Blast2GO for the pathway retrieving. All mapping and retrieving steps were performed by default setting.

### Network construction and visualization

Co-expression analysis was performed using WGCNA in order to identify the modules of highly correlated genes^49^,^50^. CV values were calculated for all genes, and those with a CV less than 0.8 across samples were not included in the WGCNA analyses. All FPKM expression values of the genes were log2 transformed, and any log2 FPKM values less than 1 were set to 0. An R version 3.0.1 (R Development Core Team, 2013) implementation of the WGCNA package was used to identify gene modules^51^ with parameters β and treecut of 20 and 0.9, respectively. All other parameters were set with the default values. Eigengenes, the average normalized gene expressions for a module, were calculated for each gene co-expression module. Eigengenes are the first principal component analysis of the normalized expression values of all genes in a module, and they represent the average normalized gene expression for a module^52^. Cytoscape v3.0.1^53^ was used to visualize the networks and Photoshop was used to edit the images.

### Real-time PCR analysis of TNF mRNA expression

The quantitative real-time PCR amplification was carried out in a total volume of 25.0 μL, containing 2.0 μL 100 × diluted cDNA, 12.5 μL 2 × SYBR Premix Ex Taq (Applied Biosystems, USA), 0.5 μL of each primers (10 mmol L^−1^) and 10.0 μL DEPC-water. The expression levels of three TNF genes (CGI_10005109, CGI_10005110 and CGI_10006440) were determined after the neurotransmitter stimulation. Three PCR products with the length of 206, 168 and 114 bp were amplified with primers designed on the basis of full cDNA sequence of the three TNFs (Table 3), respectively. Two primers for EF gene were used to amplify a fragment of 94 bp as an internal control to verify the successful reverse transcription and to calibrate the cDNA template. The SYBR Green real-time PCR assay was carried out in an ABI PRISM 7500 Sequence Detection System (Applied Biosystems) as described by Zhang *et al.*^54^. After the PCR program, dissociation curve analysis of amplification products was performed to confirm that only one PCR product was amplified and detected. All data were given in terms of relative mRNA expression.

### Nuclear and cytoplasmic protein extraction and western blotting analysis

Haemolymph from twenty individuals in each group was pooled together as one sample and centrifuged at 800 g at 4 °C for 10 min to harvest the haemocytes. The nuclear and cytoplasm proteins in haemocytes were extracted using Nuclear and Cytoplasmic Protein Extraction Kit (Biyotime) according to the protocol. The concentration of obtained protein was quantified by BCA method, and then stored at −80 °C before use.

After SDS-PAGE, the proteins were electrophoretically transferred onto two 0.45 mm pore nitrocellulose membranes at 14 mA and 20 mA for 1 h, respectively. The membranes were blocked with PBST (PBS with 1% Tween-20) containing 5% skim milk powder at 37 °C for 1 h, and incubated with polyclonal antibodies anti-rCgRel and anti-rCgp65 (1:400, prepared previously) respectively at 4 °C overnight, following by washing three times with PBST. Antibody binding was detected with goat-anti-rat Igalkaline phosphatase conjugate (Abcam) diluted 1:4000 in PBS at 18 °C for 3 h, and washed three times with PBST. Protein band was stained with Western Lightening Plus-ECL Kit (PerkinElmer) according to the protocol, and the reaction was stopped by washing with distilled water. Rats’ non-immune serum was used as negative control.

### The determination of haemocyte apoptosis and phagocytosis

The apoptosis index (AI) of oyster haemocytes was determined according to the manual of Annexin V-FITC Apoptosis Detection Kit (KeyGEN, China). The collected haemocytes (10^6^ in total) were washed twice by sterile seawater, and resuspended by 195 μL Binding Buffer. The haemocyte resuspension was incubated with 5 μL Annexin V-FITC in dark for 10 min, and resuspended with 190 μL Binding Buffer after centrifugation at 1000 *g* for 5 min. After incubated with 10 μL Propidium Iodide at 0 °C in dark for 15 min, the haemocytes resuspension were transferred into a polystyrene round-bottom tube and the AI was determined by flow cytometry (BD FACS Aria II SORP). The average AI of three tubes was calculated as Equation 1:


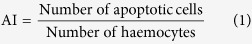


As for the assay of phagocytic rate (PR), the rinsed haemocytes were adjusted to 1.0 × 10^6^ cell mL^−1^ with sterile seawater, and 5 μL *Vibrio anguillarum* dyed by FITC (1.0 × 10^9^ CFU mL^−1^) was added to 500 μL haemocytes resuspension. After incubation at 18 °C for 1 h, 10 μL Trypan Blue (100 mg mL^−1^) was added into the mixture, and 400 μL haemocyte resuspension was transferred to a polystyrene round-bottom tube and the PR was determined by flow cytometry (BD FACS Aria II SORP). The average PR of three tubes was calculated as Equation 2:


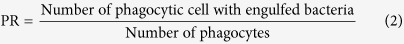


Three parallel replicates were performed in each phagocytosis and apoptosis assay.

### Statistical analysis

Statistical analysis was performed and all data were given as Means ± S.D. Statistical significance was determined by two-tailed Student’s t-test, or by one-way analysis of variance (ANOVA) followed by S-N-K post hoc test for multiple comparisons. Statistically significant difference was designated at *p* < 0.05 and extremely significant at *p* < 0.01.

## Additional Information

**How to cite this article**: Liu, Z. *et al.* The simple neuroendocrine-immune regulatory network in oyster *Crassostrea gigas* mediates complex functions. *Sci. Rep.* **6**, 26396; doi: 10.1038/srep26396 (2016).

## Supplementary Material

Supplementary Information

## Figures and Tables

**Table 1:** The overview of RNA-Seq.

Barcode ID	Treatment	Raw reads	Mapping rate	Mapping reads	Uniq mapping
37	PBS	58,871,902	39.16%	23,053,323	73.17%
38	PBS + TNF	37,358,152	45.65%	17,053,679	69.63%
39	LPS + PBS	66,568,736	39.33%	26,179,392	70.02%
40	LPS + ENK	71,228,361	37.21%	26,501,937	69.41%
41	LPS + ACh	54,453,806	35.53%	19,346,160	69.39%
42	LPS + NE	51,611,051	46.02%	23,752,891	69.81%
43	LPS + ENK + ACh	36,953,728	44.52%	16,453,106	69.65%
44	LPS + ENK + NE	56,717,161	41.97%	23,805,118	68.63%
45	PBS	152,434,137	54.10%	82,464,730	72.74%
46	PBS + TNF	21,893,548	58.61%	12,830,797	70.31%
47	LPS + PBS	58,624,191	55.52%	32,549,666	69.95%
48	LPS + ENK	66,044,178	32.80%	21,661,138	69.92%
49	LPS + ACh	55,947,854	41.13%	23,010,392	70.90%
50	LPS + NE	37,239,611	56.59%	21,072,681	69.68%
51	LPS + ENK + ACh	153,850,540	56.03%	86,193,897	71.03%
52	LPS + ENK + NE	39,706,367	59.24%	23,521,174	68.74%

**Table 2:** The network molecules.

Pathway	Gene id	Description	Gene list	network
TNF	CGI_10005109	TNF	LPS + ACH↑	blue
TNF	CGI_10005110	TNF	LPS + ENK↑	yellow
TNF	CGI_10006440	TNF	LPS + ENK↑	turquoise
TNF	CGI_10024309	TRAF-1	LPS + ENK↓, TNF + ENK + NE↓	yellow
TNF	CGI_10010206	TRAF-3	LPS + ACH↓	blue
TNF	CGI_10023156	TRAF-3	LPS + ENK↓	turquoise
TNF	CGI_10027979	TRAF-6	LPS + ENK↑, LPS + NE↓	turquoise
NF-κB	CGI_10016949	NF-κB	LPS + NE↑	brown
NF-κB	CGI_10005825	IκB	LPS + ENK↑	turquoise
NF-κB	CGI_10017654	IκB	LPS + ENK↑, LPS + ENK + NE↓	yellow
NF-κB	CGI_10016955	IKK	LPS + ENK↑, LPS + NE↑, LPS + ENK + ACH↑	blue
AP-1	CGI_10012291	AP-1	LPS + ENK + ACH↓	grey
AP-1	CGI_10018951	AP-1	LPS + ENK↑	yellow
AP-1	CGI_10023787	AP-1	LPS + ENK↑	turquoise

**Table 3:** Sequences of the primers used in the experiment.

Primer	Sequence (5′-3′)	Sequence information
P1 (forward)	CGCAATGGTCGCTTGGTGGTC	Real-time TNF (CGI_10005109) primer
P2 (reverse)	CGTAGGGGCGGAAGGTCTCG	Real-time TNF (CGI_10005109) primer
P3 (forward)	CAACGGTCTAACTTACCATCCAAAC	Real-time TNF (CGI_10005110) primer
P4 (reverse)	TGGTGGTAGATAAAATGGGACAGTG	Real-time TNF (CGI_10005110) primer
P5 (forward)	ATTGGAGCACCTGGAGGATAAG	Real-time TNF (CGI_10006440) primer
P6 (reverse)	CAGTCTTCCGTGCTGGTATTTC	Real-time TNF (CGI_10006440) primer
P7 (forward)	ATCCTTCCTCCATCTCGTCCT	Real-time EF primer
P8 (reverse)	GGCACAGTTCCAATACCTCCA	Real-time EF primer
P9 (forward)	ATGAATCTACCACCGATCGAAGG	CgTNF-1 specific primer
P10 (reverse)	TTTTGGCGTCTTCAAGCTGTGA	CgTNF-1 specific primer
M13-47	CGCCAGGGTTTTCCCAGTCACGAC	pMD18-T simple vector primer
RV-M	GAGCGGATAACAATTTCACACAGG	pMD18-T simple vector primer
P11 (forward)	GGCCACGCGTCGACTAGTACT_17_	Oligo(dT)-adaptor
