# Supplementary material for: The simple neuroendocrine-immune regulatory network in oyster Crassostrea gigas mediates complex functions
Source: Sci Rep. 2016 May 19;6:26396. doi: 10.1038/srep26396 (PMC4872224; doi:10.1038/srep26396)
Supplement: Supplementary Information [file srep26396-s1.pdf]

The simple neuroendocrine-immune regulatory network in oyster *Crassostrea gigas* mediates complex functions

Zhaoqun Liu [a,c,#], Lingling Wang [a,b,#], Zhi Zhou [a], Ying Sun [a,c], Mengqiang Wang [a], Hao Wang [a], Zhanhui Hou [a], Dahai Gao [a], Qiang Gao [a], Linsheng Song [b,*]

[a] Key Laboratory of Experimental Marine Biology, Institute of Oceanology, Chinese Academy of Sciences, Qingdao 266071, China

[b] Key Laboratory of Mariculture & Stock Enhancement in North China's Sea, Ministry of Agriculture, Dalian Ocean University, Dalian 116023, China.

[c] University of Chinese Academy of Sciences, Beijing 100049, China

[#]These authors contributed equally to this study.

*Correspondence to:

Dr. Linsheng Song

Dalian Ocean University, Dalian 116023, China.

Tel: + 86 411 84763173

Email address: lshsong@dlou.edu.cn

Figure S1. The full-length gels of the translocation of 2 oyster NF-κB molecules in haemocytes nuclei and cytoplasm. All the gels have been run under the same conditions, and the cropping lines are indicated with the red square frames.

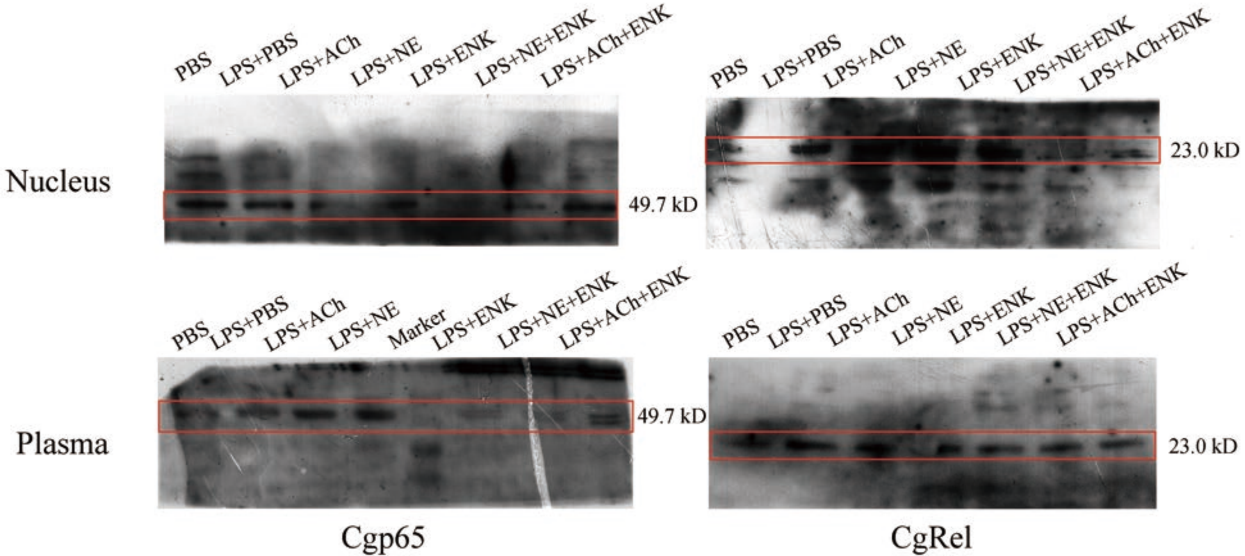

PBS
LPS+PBS
LPS+ACh
LPS+NE
LPS+ENK
LPS+NE+ENK
LPS+ACh+ENK
Nucleus
49.7 kD
23.0 kD
Marker
Plasma
Cgp65
CgRel
